# PADI6: What we know about the elusive fifth member of the peptidyl arginine deiminase family

**DOI:** 10.1098/rstb.2022.0242

**Published:** 2023-11-20

**Authors:** Jack P. C. Williams, Louise J. Walport

**Affiliations:** ^1^ The Francis Crick Institute, London, NW1 1AT, UK; ^2^ Imperial College of Science Technology and Medicine, London, W12 0BZ, UK

**Keywords:** protein citrullination, fertility, early embryo development, arginine deiminase, embryonic genomic activation, post-translation modifications

## Abstract

Peptidyl arginine deiminase 6 (PADI6) is a maternal factor that is vital for early embryonic development. Deletion and mutations of its encoding gene in female mice or women lead to early embryonic developmental arrest, female infertility, maternal imprinting defects and hyperproliferation of the trophoblast. PADI6 is the fifth and least well-characterized member of the peptidyl arginine deiminases (PADIs), which catalyse the post-translational conversion of arginine to citrulline. It is less conserved than the other PADIs, and currently has no reported catalytic activity. While there are many suggested functions of PADI6 in the early mouse embryo, including in embryonic genome activation, cytoplasmic lattice formation, maternal mRNA and ribosome regulation, and organelle distribution, the molecular mechanisms of its function remain unknown. In this review, we discuss what is known about the function of PADI6 and highlight key outstanding questions that must be answered if we are to understand the crucial role it plays in early embryo development and female fertility.

This article is part of the Theo Murphy meeting issue ‘The virtues and vices of protein citrullination’.

## 1. Introduction

**Figure 1.**
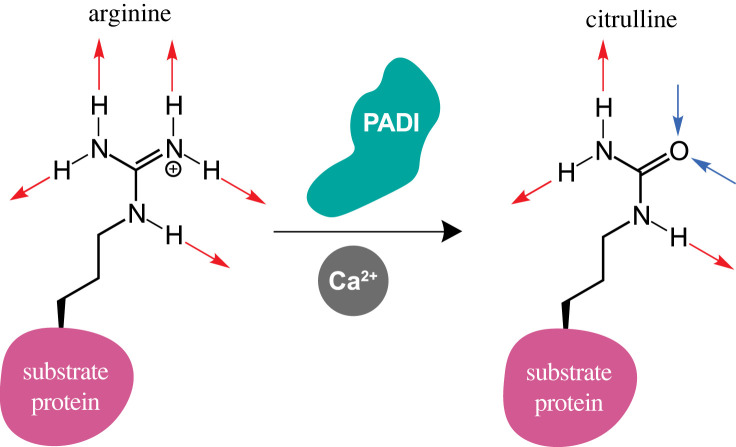
Peptidyl arginine deiminase (PADI) catalyses post-translational conversion of arginine to citrulline in the presence of Ca^2+^. Red arrows, H-bond donor; blue arrows, H-bond acceptor.

The peptidyl arginine deiminases (PADIs or PADs) catalyse citrullination, the fundamental yet poorly understood post-translational conversion of arginine to citrulline (figure 1) [1]. The conversion of the positive, coded arginine side chain to the neutral, non-coded citrulline can cause significant structural and functional changes in substrate proteins. Consequently, absence of, or aberrant PADI activity has been associated with a range of pathologies including rheumatoid arthritis, multiple sclerosis, various cancers and female infertility [2–7].

In mammals, there are five PADIs (PADIs 1–4 and PADI6), each with a unique tissue expression and substrate profile [1,8–10]. Of the PADI family, PADI6 was the last to be identified and is the least well-characterized. Predominantly expressed in the oocyte and early embryo, PADI6 is crucial for early embryonic development and female fertility in both humans and mice [7,11–24]. However, the molecular mechanisms responsible for this role are unclear, and notably there is currently no reported catalytic activity of PADI6.

Structural and biophysical techniques have been implemented extensively in the functional studies of human PADIs 2 and 4 (hPADI2 and hPADI4), providing valuable insight into the mechanisms of calcium activation and substrate deimination [25,26]. However, since the discovery of the mouse *Padi6* gene in 2003 (previously termed ePAD, for embryo and egg abundant PAD-like protein) [10] and identification of the human orthologue later that year [27], little structural or biophysical characterization has been reported. Interestingly, while human PADI6 (hPADI6) shares sequence homology with the other family members, it is much less conserved with hPADIs 1–4 (average conservation: 44%) than they are with each other (average conservation: 54%).

Despite its fundamental role in early embryonic development, PADI6 remains under-characterized. This review aims to summarize what is known about PADI6 and to highlight some key questions that are yet to be answered about the elusive fifth member of the PADI family. First, evidence supporting a crucial role for PADI6 in embryo development and female fertility is summarized. Next, the known biochemistry of PADI6 is covered, along with insights that we can derive from the sequence conservation between PADI6 and the other PADIs. Finally, the experimental work aimed at understanding the function of PADI6 in early embryo development is detailed, summarizing the current theories and highlighting key outstanding questions.

## 2. PADI6 is vital for early embryo development and fertility

**Figure 2.**
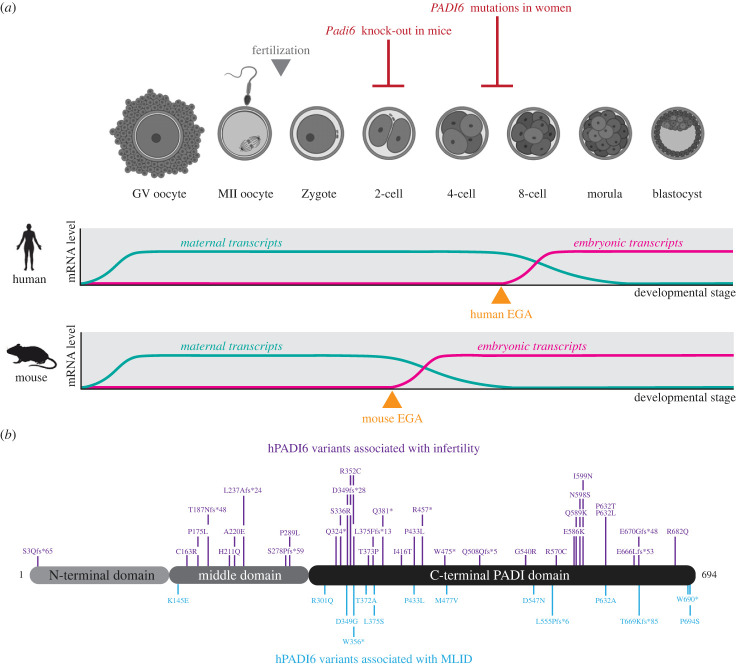
Early embryo development and PADI6. (*a*) Schematic of mammalian oocyte and preimplantation embryo development and dynamic changes in mRNA levels. The developmental arrest times caused by the absence of PADI6 in mice by knock-out, or in humans by loss-of-function mutation, and the timing of embryonic genome activation (EGA) in mice (2-cell stage) and humans (4- to 8-cell stage) are indicated. Embryo cartoons created with BioRender.com. GV as germinal vesicle. (*b*) Pathogenic PADI6 variants reported in women divided into those that are associated with infertility (purple) or pregnancy complications and offspring with MLID (blue). MLID, multi-locus imprinted disorders.

PADI6 is vital for early embryonic development in humans and mice. As a maternal effect gene, it is provided to the maturing oocyte throughout oogenesis, accumulating large pools of both mRNA and protein [28,29]. Like other maternal effect genes, the absence of PADI6 in the maturing oocyte and early embryo has drastic consequences (figure 2*a*). The importance of PADI6 was revealed in 2007 by Coonrod and co-workers, who demonstrated that PADI6 knock-out female mice were infertile, with embryos arresting at the 2-cell stage [7]. Sperm–egg binding, fusion, sperm decondensation and pronuclear formation all occur normally in PADI6 knock-out mouse oocytes, suggesting PADI6 has no role in fertilization. It was not until 2016 that a similar human PADI6 phenotype was reported after whole-exome sequencing revealed homozygous premature *PADI6* nonsense mutations and compound heterozygous mutations in the genomes of infertile women [11]. Since 2016, 44 pathogenic *PADI6* mutations, in a total of 35 women, have been reported with variations in observed phenotypes corresponding to the severity and zygosity of the mutations (figure 2*b*; tables 1 and 2).

**Table 1.** Human PADI6 variants reported in infertile women.

PADI6 variants	zygosity	phenotype	reference
S3Qfs*65/C666Lfs*53	compound heterozygous	early embryonic arrest	[20]
C163R	homozygous		[24]
T187Nfs*48/T187Nfs*48	homozygous		[20]
H211Q/L237Afs*24	compound heterozygous		[20]
H211Q/E670Gfs*48	compound heterozygous		[11]
A220E/A220E	homozygous		[20]
L237Afs*24/S336R	compound heterozygous		[20]
S278Pfs*59/S278Pfs*59	homozygous		[15,18]
P289L/P632L	compound heterozygous		[13]
Q324*/G540R	compound heterozygous		[11]
D349fs*28/P433L	compound heterozygous		[20]
R352C	homozygous		[20]
T373P/R570C	compound heterozygous		[15]
L375Ffs*13	homozygous		[13]
Q381*	homozygous		[11]
I416T	homozygous		[20]
R457*	homozygous		[30]
W475*	homozygous		[24]
S508Qfs*5	homozygous		[15]
G540R	homozygous		[20]
G540R/P632T	compound heterozygous		[20]
E586K	homozygous		[20]
Q589K/E670Gfs*48	compound heterozygous		[20]
I416T / E670Gfs*48	compound heterozygous		[31]
N598S/R682Q	compound heterozygous	six miscarriages, one hydatidiform mole	[12]
I599N	homozygous	three miscarriages, four hydatidiform moles	[19]

**Table 2.** Human PADI6 variants reported in mothers who gave birth to children with multi-locus imprinted disorders (MLID). wt, Allele encoding wild-type hPADI6 protein; SRS, Silver–Russell Syndrome; TS, Temple Syndrome; BWS, Beckwith–Weidemann Syndrome.

PADI6 variants	zygosity	phenotype	reference
K145E/wt	heterozygous	one child with SRS	[33]
R301Q/P433L	compound heterozygous	one child with SRS and TS	[34]
D349G/wt	heterozygous	one child with SRS, two children with no MLID	[33,34]
W356*/P632A	compound heterozygous	two children with BWS	[14]
T372A/W690*	compound heterozygous	two miscarriages, one premature birth, two children with BWS	[17]
L375S/D547N	compound heterozygous	one child with BWS, reproductive history not reported	[34]
M477V/P694S	compound heterozygous	one miscarriage, one child with BWS, one child with no BWS	[14]
D547N/L555Pfs*6	compound heterozygous	nine miscarriages, one child with BWS	[22]
T669Kfs*85/wt	heterozygous	one child with BWS, two children with no BWS	[14,33]

### (a) *PADI6* mutations in women

Whole-exome sequencing of 26 infertile women has led to the identification of 32 pathogenic *PADI6* mutations that, when homozygous or compound heterozygous, are associated with female infertility (table 1; figure 2*b*). Embryos from these women arrest predominantly at the 4- to 8-cell stage [11,13,15,16,18,20,23,24,30,31]. While this is at a later stage than in the mouse, intriguingly in each species this coincides with the timing of EGA (EGA occurs at the 2-cell stage in mice and between the 4- and 8-cell stage in humans), suggesting a potential role in EGA, discussed in §4b [32]. Of these 32 mutations, 13 produce a truncated or frameshifted protein. The remaining 19 missense mutations are likely to be involved in crucial folding interaction, resulting in a misfolded and ultimately degraded protein, or in key protein–protein interactions (PPIs). The deleterious effect of several of these mutations has been confirmed by immunofluorescence and western blot analysis of oocytes and embryos from patients possessing pathogenic mutations. For example, immunofluorescence analysis of oocytes from patients either possessing homozygous Q381*, compound heterozygous H211Q/E670Gfs*48 or homozygous L375F fs*13 confirms an absence of PADI6 protein compared to oocytes from a healthy patient [11,13].

While embryos from 24 of the infertile women are associated with early embryonic developmental arrest, two women were noted to have extended pregnancies. However, in both women these pregnancies ended prematurely with miscarriage or the formation of a hydatidiform mole (HM), characterized by hyperproliferation of the trophoblast. One patient with compound heterozygous N598S/R682Q, had six reported pregnancies, ranging from 7 to 10 weeks in duration, of which five ended with non-molar miscarriages and one in the formation of a HM [12]. The other patient, with a homozygous mutation producing I599N PADI6, had a total of seven pregnancies that ended with three miscarriages and the formation of four HMs. Two of these molar pregnancies reached as late as 15 and 32 weeks gestation [19]. The extended duration and altered phenotype of these pregnancies have been attributed to the differences in mutation severity, specifically the effects of missense mutations versus premature truncations.

Of the remaining women, broader and less pronounced phenotypes are observed (table 2). Nine reported women possessing a total of 14 pathogenic *PADI6* mutations have been able to have children, though often with a high incidence of pregnancy complications including recurrent miscarriages [14,17,22,33,34]. For example, a compound heterozygous woman producing the T372A/W690* PADI6 variants had a reported three failed pregnancies and two children, both of whom were born with multi-locus imprinting disorders (MLID) [17]. Of the three failed pregnancies, the first child was born at gestational week 33 + 3 and died immediately after birth. The second and third pregnancies both resulted in miscarriages; the first fetus possessed an aneuploid karyotype, while the second possessed a normal karyotype. Interestingly, MLID phenotypes have been observed in at least three heterozygous patients, suggesting a dominant effect [14]. Overall, the disparity between pregnancy phenotypes demonstrates the broadness and complexity of *PADI6* mutations and the gene's overall function in embryo development.

### (b) *PADI6* mutations and multi-locus imprinting disorders

As highlighted above, several *PADI6* mutations have been reported in fertile women, however their children often have MLID. MLID are defined as imprinting disorders with alterations in multiple imprinted loci. Notable examples include Beckwith–Wiedemann syndrome and Silver–Russell syndrome [33,35,36], both of which have been associated with PADI6. Imprinting disorders are congenital conditions that result from alterations in the function or regulation of imprinted genes [35]. Genomic imprinting refers to the parent-of-origin-dependent monoallelic expression of a subset of autosomal genes, with such genes termed imprinted genes [37]. Around 100 human genes are predicted to be imprinted [38].

A variety of epigenetic factors have been reported to be involved in this parent-of-origin expression, predominantly differential DNA methylation between alleles, which results in different accessibility of gene promoters to transcription factors [36,37]. The epigenetic characteristics associated with patients suffering from MLID include either hypo- or hypermethylation at multiple imprinted loci and because the alterations occur in multiple locations across the genome, MLID are most commonly caused by deletions or mutations in the genes of *trans*-acting factors [33]. For comprehensive reviews on imprinting, its regulation and associated disorders, see [36,37,39].

The maternal imprinted signature is established during oogenesis, finished in the mature oocytes and maintained through the global demethylation that occurs after fertilization [36,40]. Methylation sequencing of PADI6-attributed MLID patients showed loss of methylation predominantly at specific imprinted loci, with unique profiles for each patient tested [14,17]. The exact role of PADI6 in maternal imprinting is not understood and, at the time of writing, the available data are limited. In the future, transcriptomic, methylomic and chromatin accessibility analysis of patients suffering from PADI6-attributed MLID could be highly valuable in understanding the molecular mechanisms of not only PADI6, but other *trans*-acting factors implicated in MLID.

### (c) PADIs in the early embryo

Like other maternal effect genes, *Padi6* mRNA and protein are abundant in the oocyte and early embryo, with negligible expression in most other tissues [10,41]. A large-scale comparative proteomic experiment conducted by Gao and co-workers, taking mouse embryos from each developmental stage from zygote to blastocyst [42], identified mPADI6 as the most abundant protein in the early mouse embryo, with the next most abundant being DNMT1. Using tandem-mass-tag (TMT) labelling, the relative protein level of PADI6 between developmental stages was interrogated, with PADI6 protein level remaining abundant and relatively steady until the blastocyst stage, when a reduction was seen. A parallel single-cell RNA-seq (scRNA-seq) experiment conducted in the same study found that the RPKM (reads per kilobase per million reads mapped) values for *Padi6* were high at the zygote stage and then dropped off from the 2-cell stage onwards, which is typical of the mRNA profile of a maternal effect gene being degraded post-EGA [28,43].

Data describing the presence of PADIs 1–4 in the early embryo, however, are conflicting. Gao and co-workers [42] failed to identify any peptides from PADIs 1–4 at any stage of development, out of a total of 3767 detected proteins. Similarly, negligible RPKM values were observed for PADIs 1–4 from the zygote to blastocyst stage, indicating low to no expression. However, in contrast to this proteomic and RNA-seq data, immunofluorescence-based experiments conducted by Coonrod and co-workers detected PADIs 1–4 as well as PADI6 throughout mouse early embryo development [44].

In 2022, Zhang and co-workers adapted Ribo-seq, a technique to profile mRNA translation on the ribosome, for small sample input quantities, coining Low Input Ribosome sequencing (LiRibo-seq) [45]. They used LiRibo-seq to provide a temporal insight into protein expression throughout development by measuring the mRNA translation profile of the early mouse embryo. Two translational spikes were seen for *Padi6*, first at the metaphase II arrested oocyte stage (the earliest timepoint measured) and next at the 4-cell embryo stage. By the 4-cell stage the embryonic genome has been activated, raising the question of whether, at the 4-cell stage translational spike, *Padi6* is being transcribed and translated from the embryonic genome after its activation or translated from the maternal mRNA pool. As with the previously described scRNA-seq data, PADIs 1–4 yielded negligible RPKM values. Together, these data demonstrate that PADI6 is an abundant and dynamically regulated protein during mouse early embryonic development and is likely the dominant arginine deiminase expressed at this early stage of development.

## 3. The biochemistry of PADI6

This section summarizes the key differences that set PADI6 apart from the other PADIs, along with the current research characterizing PADI6 catalytically and structurally.

### (a) Active site conservation

**Figure 3.**
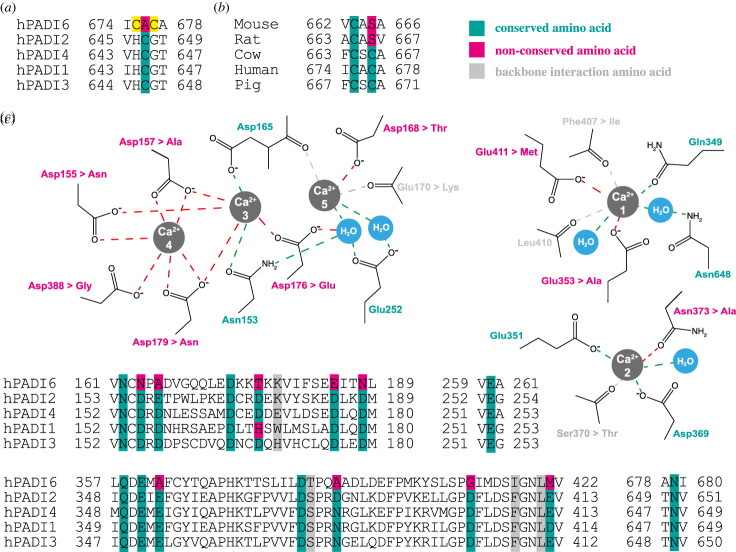
Sequence conservation of PADI6. (*a*) Sequence alignment of the human PADIs centred on the key catalytic cysteine residue. Yellow shading = potential hPADI6 key catalytic cysteine residues. (*b*) Sequence alignment of PADI6 from mouse, rat, cow, human and pig *Padi6* centred on the key predicted active site cysteine residues. (*c*) Differences in the hPADI6 and hPADI4 calcium binding pockets mapped onto the assigned hPADI4 calcium binding schematic, adapted from [25]. All alignments were produced using Clustal Omega [46].

PADIs are cysteine hydrolase enzymes: a key nucleophilic cysteine residue, two aspartic acid residues and a histidine are required for catalytic activity [25]. While the aspartic acid and histidine residues are conserved across all hPADIs, the key nucleophilic cysteine residue is not (figure 3*a*). Where hPADIs1–4 possess the cysteine, hPADI6 has an alanine flanked by two cysteine residues. This raises numerous questions, notably: if hPADI6 has catalytic activity, which of the two cysteines is key for citrullination? Furthermore, given the proximity of the two cysteine residues, do they participate in disulfide bond formation? And if so, could the catalytic activity of hPADI6 be regulated by redox chemistry? Towards answering the first questions, sequence alignments of PADI6 show that only the first of the two cysteine residues is conserved across multiple species, with the second being substituted by a serine in mice and rats (figure 3*b*). This implies that if PADI6 is capable of catalysing citrullination, the key nucleophilic cysteine is likely the first of these two.

### (b) Calcium binding

PADIs 1–4 each have between 4 and 6 binding sites for Ca^2+^ ions, and high calcium concentrations are required for *in vitro* activity [25,26,47]. Excluding backbone interactions, only 7 out of 16 of the hPADI4 residues directly involved in calcium binding are conserved in hPADI6 (figure 3*c*). To date, no research has been conducted to directly characterize the ability of hPADI6 to bind calcium. However, given the lack of conservation in binding residues it appears unlikely that hPADI6 will bind, and by extension, be activated by calcium. For example, it has been reported that switching just one of the calcium binding residues in a pocket to alanine can dramatically reduce the catalytic activity of hPADI4 in the presence of calcium [25]. This potential lack of calcium-dependent activation could be explained from an evolutionary perspective; immediately after fertilization, a large calcium influx into the newly fertilized zygote induces a cascade of further developmental changes [40]. To avoid PADI6 function being affected by this large transient increase in calcium concentration, one strategy would be genetic divergence of PADI6 such that it is not activated by calcium. Biophysical characterization of the calcium binding capacity of hPADI6 is necessary to support this hypothesis.

### (c) Catalytic activity

Recombinant mPADI6 yielded no detectible *in vitro* activity in the colour developing reagent (COLDER) assay when performed at high calcium concentrations using the pan PADI substrate, *N*-α-benzoyl-l-arginine ethyl ester (BAEE) [48]. While this could reflect a general lack of catalytic activity, it could also reflect use of the wrong substrate or activating conditions. Given that it is unlikely that PADI6 binds calcium, the calcium provided in the assay is unlikely to be activating. Calcium binding induces structural changes in the PADI catalytic domain, opening the active site for substrate binding [25]. It is conceivable that such changes could also be induced through a protein–protein interaction (PPI) with an alternative activating cofactor. Indeed, the calcium concentrations necessary to achieve citrullination by PADIs 1–4 *in vitro* are far higher than the maximum physiological intracellular calcium concentrations reported [49], leaving an unanswered question as to how other PADIs are activated *in vivo*.

It has also been shown that PADIs 2–4 form homodimers, and in the case of PADI4 this dimerization both increases catalytic activity and calcium binding cooperativity, although the monomer is still catalytically active [50]. Whether PADI6 forms similar homodimers is unclear. The only published structural characterization of PADI6 was reported in Taki *et al.* [48], who expressed and purified recombinant mouse PADI6 (mPADI6). Studying this by chemical cross-linking, mPADI6 appeared to form higher-order oligomeric structures, with the largest being a hexamer. Further characterization of this possible oligomerization, and identification of interacting proteins, will be crucial both for uncovering potential activation mechanisms and also for understanding the overall function of PADI6.

### (d) Phosphorylation and regulation by 14–3–3 proteins

In addition to PPIs, post-translational protein modifications, such as phosphorylation, have the potential to modify protein function and activity. For example, 14–3–3 proteins can act as regulatory factors through their interaction with specific phosphorylated sequences (motif: ArgXXpSer or ArgXXXpSer motifs) in target proteins [51–53]. Using an antibody specific to the phosphorylated 14–3–3 binding motif, Rose *et al.* showed that PADI6 was phosphorylated and interacted with 14–3–3 proteins at the mature egg and 2-cell embryo stages (no staining was observed in immature mouse oocytes) [54]. Specific phosphorylation sites were not identified. In subsequent work, Rose *et al.* attempted to structurally characterize the PADI6/14–3–3 interaction [55]. Using an *in silico* workflow, two canonical and a further six non-canonical possible 14–3–3 binding residues were identified, and the corresponding phosphorylated peptides synthesized. Two of the peptides, centred at Ser10 (canonical) and Ser446 (non-canonical), interacted with 14–3–3*σ* by fluorescence polarization. Structural characterization by X-ray crystallography revealed both peptides docking into the expected 14–3–3 binding pocket. It is not clear which, if either, of these two serines is the endogenously phosphorylated and 14–3–3-interacting residue, however, and the relevant kinase and overall biological function of PADI6 phosphorylation remain unknown.

## 4. What are the functions of PADI6?

**Figure 4.**
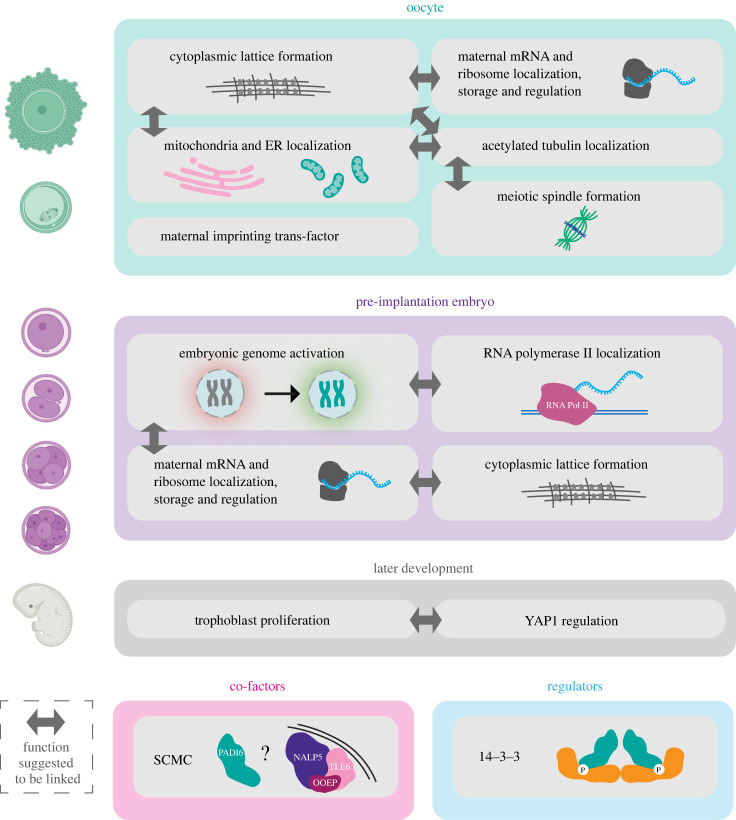
Suggested functions of PADI6 in the mouse and human oocyte and/or embryo development categorized by developmental time period, with suggested cofactors and regulators. NALP5, OOEP, TLE6: proteins encoded by maternal effect genes and conserved between species in the core subcortical maternal complex (SCMC). YAP1: a downstream modulator of the Hippo signalling pathway; SCMC, subcortical maternal complex. Embryo cartoons created with BioRender.com.

PADI6 has many proposed functions based on experimental observations in the oocyte and early embryo. In this section, the published experimental work into the possible functions of PADI6 in mouse early embryonic development is reviewed, along with any parallel data in the human oocyte or early embryo. A summary of the suggested functions and potential relationships between them is displayed in figure 4.

### (a) Does PADI6 citrullinate proteins in the early embryo?

The detection of citrullinated proteins in a cellular context still poses a challenge to the PADI field, and there are currently few reagents that can identify citrullination in a site-agnostic manner using low input samples. Upon immunohistochemical staining of wt and PADI6 knock-out ovaries with an antibody specific to citrullinated histone 4 peptide (His4Cit3), a decrease in oocyte staining of ovarian sections was detected in the PADI6 knock-out ovaries [7]. Contrastingly, in a later work, immunofluorescence analysis of wt and PADI6 knock-out oocytes with a range of citrullinated peptide antibodies (His3Cit2 + 8 + 17, His4Cit3, His3Cit26) showed no difference in staining between wt and PADI6 knock-out samples [56]. This discrepancy in experimental data highlights the limitations of antibody-based strategies for interrogating protein citrullination and leaves the answer as to whether proteins are citrullinated in the oocyte and early embryo unclear.

As an alternative to genetic methods, and commonly used in conjunction with immunofluorescence-based strategies, PADI citrullination can be interrogated using PADI inhibitors. Cl-Amidine is a well-known covalent pan-PADI inhibitor and D-Cl-Amidine a derivative that is selective for PADI1 over PADIs 2–4 [57,58]. Treatment of zygotes with 100 µM d-Cl-Amidine resulted in a decrease in nuclear staining of 4-cell embryos with His4Cit3 and His3Cit2 + 8 + 17 antibodies [44]. Developmentally, no compound-treated zygotes reached the morula stage, compared to 75% of vehicle-treated zygotes. Similar results were observed with the pan-inhibitor Cl-amidine [56]. While Cl-amidine is known to inhibit PADIs 1–4, no data are available for its activity against PADI6. Given the apparent proteomic and transcriptomic absence of PADIs 1-4 in the early embryo, and the low specificity of Cl-Amidine towards the different PADI isoforms, it is plausible that the observed effects of Cl-amidine (and D-Cl-amidine) treatment are due to the effect of the inhibitor on PADI6. Alternatively, due to the propensity of Cl-amidine-based inhibitors to react with nucleophilic cysteine residues, it is conceivable that prolonged exposure to high concentrations of inhibitor could impact important off-targets in the early embryo, leading to the reduced developmental capacity and other phenotypes observed [58,59]. Overall, experimental data concerning possible citrullination and PADI activity in the early embryo are contrasting and further work is required to fully elucidate the true story.

### (b) Is PADI6 required for embryonic genome activation?

Embryonic arrest in the absence of PADI6 coincides with the timing of EGA in both humans and mice (figure 2*a*). It has therefore been suggested that PADI6 may play a crucial role in successful EGA. During its activation, the embryonic genome undergoes dramatic reorganization, leading to the highly regulated transcription and translation of key early embryonic proteins required for the completion of EGA and the first embryonic divisions, as well as the controlled degradation of maternal factors [60,61]. As such, mRNA transcription and translation globally, and of known key early embryonic factors, can be used as markers for successful EGA [62].

In 2-cell embryos from PADI6 knock-out mice, levels of RNA polymerase II (RNA Pol II), as measured by immunofluorescence, were decreased by approximately 20% [63]. RNA Pol II also appeared to aggregate around the nuclear envelope. Furthermore, levels of nuclear phosphorylated RNA Pol II (a proxy for active transcription) were decreased by approximately 30% compared with wt embryos. This is also seen in arrested 4-cell human embryos from women possessing the homozygous Q381* or compound heterozygous H211Q/E670Gfs*48 PADI6 variants, where a dramatic decrease in phosphorylated RNA Pol II was observed in embryos by immunofluorescence [11]. Together, these results suggest that PADI6 is important for nuclear translocation of RNA Pol II and/or its subsequent phosphorylation to the actively transcribing form. In conjunction with these data, a reduction of approximately 10% in histone H4 acetylation (a marker for transcription) was observed in the 2-cell embryos from PADI6 knock-out mice [63]. Transcription requiring complex (TRC) levels were also reduced by approximately 53% in these embryos compared to wt embryos. As TRC is among the first protein complexes expressed from the activated embryonic genome [64], this further supports a role of PADI6 in EGA. Overall EGA appears defective, though perhaps not completely inhibited, in the absence of PADI6. Further work is now required to understand the involvement of PADI6 in the activation of the embryonic genome.

### (c) Is PADI6 involved in maternal mRNA regulation at the cytoplasmic lattices?

First identified in 1989, the cytoplasmic lattices (CPLs) are a fibrillar structure unique to the oocyte and early embryo, and, like PADI6, are also not fully understood [65–68]. While there are many theories as to their role in the early embryo, their exact function and composition remain unknown. The CPLs are completely absent in PADI6 knock-out oocytes, suggesting PADI6 is crucial for their formation or maintenance [7]. Additionally, PADI6 appeared to localize to the CPLs, as shown by gold immunoelectron microscopy [10]. As fertilization occurs normally in PADI6 knock-out oocytes lacking the CPLs, these observations suggest that CPL function is required during the first embryo divisions.

In 2008, Yurttas *et al.* discovered that the CPLs possessed a ribosomal component, as a large portion of the maternal ribosomes were associated with a large supramolecular complex [63]. This association was disrupted in PADI6 knock-out oocytes, suggesting that these supramolecular complexes, likely the CPLs, act as a storage or regulation site for maternal ribosomes in the oocyte. Crucially, overall ribosomal protein levels did not differ between wild-type and PADI6 knock-out oocytes, confirming that PADI6 only has a function in ribosomal regulation and storage and not in production or maintenance. The authors also reported a reduction of approximately 50% in total protein synthesis in PADI6 knock-out embryos, although the data were not shown.

It was further noted by Kan *et al.* [69] that during Triton X-100 extraction, components associated with the CPLs were not extracted, remaining present in the sample [69]. Comparing levels of different targets after extraction with Triton X-100 in wt and PADI6 knock-out females (lacking CPLs) therefore provided a model for identifying targets that were specifically associated with the CPLs. In a follow-up to this work, it was confirmed that the CPLs also had an mRNA component, indicating that they could act as a storage site for maternal mRNA as well as for ribosomes [70]. MSY2, an mRNA binding protein, was also identified as a CPL-associated protein and has been suggested to be involved in the interaction with and storage of maternal mRNA. MSY2 localization is severely disrupted in PADI6 knock-out oocytes, suggesting that PADI6 is required for MSY2 localization either directly or potentially through the CPLs. Prior to EGA, transcription and regulation of maternal effect genes needs to be carefully regulated so that the correct transcripts are translated or degraded at the right times. Given the tight transcriptional and translational regulation required for proper EGA, it is possible that the suggested functions of PADI6 in EGA and CPL formation are intertwined, and the observed EGA defects in PADI6 knock-out embryos are due to the absence of the CPLs and mis-regulation of maternal mRNA and ribosomes.

It has been proposed that the CPLs, along with PADI6, are important for proper localization of embryonic organelles and structures, notably the meiotic spindle, mitochondria and endoplasmic reticulum (ER) [69]. When investigating the CPLs, Kan *et al.* noted that α-tubulin appeared to co-localize and interact with PADI6. It also appeared to be associated with the CPLs based on gold-immunoelectron microscopy, and the observation that a significant amount of non-microtubule α-tubulin was retained in the cytoplasm after Triton X-100 extraction. PADI6 did not co-localize with α-tubulin at the microtubules (MT) in the meiotic and mitotic spindles, although immunofluorescence analysis of α-tubulin in mature oocytes showed that meiotic spindle MTs were shorter and had a wider equatorial spindle diameter in PADI6 knock-out oocytes compared to wt. Throughout oocyte maturation, organelles, including the mitochondria and ER, undergo dramatic reorganization mediated by MTs [71,72]. This reorganization was significantly disrupted in PADI6 knock-out germinal vesicle (GV) oocytes, as shown by fixed and live immunofluorescence imaging of the mitochondria and ER. Acetylation of α-tubulin has been linked with MT stabilization and more importantly shown to be involved in the MT trafficking of ER and potentially mitochondria in somatic cells [73–75]. Levels of cytoplasmic acetylated α-tubulin were reported to be decreased in PADI6 knock-out GV and MII oocytes [69]. No difference was observed in spindle acetylated α-tubulin between wt and PADI6 knock-out oocytes. These data indicate a possible function of PADI6/CPL in α-tubulin and MT-mediated oocyte organelle localization.

### (d) Is PADI6 a member of the subcortical maternal complex?

The subcortical maternal complex (SCMC) is a complex of at least three proteins, encoded by maternal effect genes, localized to the subcortex of early embryos [76–78]. The complex has been identified in both mice and humans, with the core proteins conserved between the species (NALP5, OOEP and TLE6 in humans). At least four further proteins (KHDC3L, ZBED3, NLRP2 and NLRP4F) have been reported to interact and participate with the complex [79–82]. When OOEP, a core member of the complex, was immunoprecipitated from mouse ovarian lysates, mass spectrometry analysis revealed that six mPADI6 peptides covering 11% of the protein were present in the immunoprecipitated fraction, suggesting a potential interaction between PADI6 and the SCMC [78]. No experimental work has been conducted to confirm this relationship; however, multiple lines of evidence further point towards a strong relationship between the two. Like PADI6, the SCMC proteins are vital for early embryonic development and loss of any member results in a similar phenotype to PADI6 knock-out, notably early embryonic developmental arrest [81,83–85]. Additionally, like PADI6, numerous SCMC gene mutations have been reported in either infertile women or women with fertility issues and/or in children with MLID, suggesting the function of PADI6 in maternal imprinting is also linked with the SCMC [33]. The CPLs are also absent in mouse oocytes missing core SCMC components MATER (NALP5 in humans) and OOEP, and it appears that PADI6 also localizes to the subcortex of mouse oocytes and early embryos [63,85], although observed localization appears dependent on fixation and permeabilization procedures [86].

While the exact function of the SCMC is still not fully understood, it is known to be required for the formation of the F-actin meshwork in oocytes and embryos [87]. This F-actin meshwork controls spindle positioning as well as being involved in mitochondrial and ER distribution. As PADI6 absence is associated with spindle, mitochondria and ER localization defects, as discussed in §4c, it is likely that PADI6 is also involved in this function. Given the absence of CPLs when PADI6 or SCMC proteins are knocked out, and the localization of both to the lattices, it has been theorized that PADI6, along with the SCMC, makes up an integral component of the CPLs, and furthermore, it is PADI6, along with the SCMC and CPLs, that is required for proper organelle localization.

### (e) Does PADI6 play a role in regulation of trophoblast cell migration–invasion?

As mentioned in §2a, *PADI6* mutations can result in a higher incidence of HM formation during pregnancy, which is linked to trophoblast cell proliferation and invasion. To characterize the relationship between PADI6 and trophoblast dysfunction, Qian and co-workers targeted YAP1, a downstream modulator of the Hippo signalling pathway that has been implicated as a key factor in proper trophoblast function [88]. PADI6 and YAP1 appeared to co-localize in the nucleus of cytotrophoblast cells of villi tissues from healthy and HM placentas and in all four human trophoblast cell lines studied. Additionally, PADI6 and YAP1 appeared to interact by co-immunoprecipitation. YAP1 expression was decreased in both PADI6 knock-out HTR-8SVneo (healthy trophoblast) and JAR (choriocarcinoma) cell lines and upregulated in cell lines overexpressing PADI6. Upregulation of PADI6 also appeared to promote migration and invasion of both cell lines in wound healing and Transwell assays, as well as promoting cell cycle progression, proliferation and apoptosis. Both F-actin and β-tubulin also appeared significantly upregulated in the PADI6 overexpressing cell lines, potentially affecting cellular cytoskeletal dynamics and cell migration. Together these findings are the first experimental data highlighting a potential role of PADI6 in trophoblast proliferation and metastasis through YAP1. However, more work is required to understand the exact nature of the relationship *in vivo*. A causative link between improper maternal imprinting and trophoblast proliferation and HM formation has been proposed, suggesting that the role of PADI6 in both pathologies could be correlated [36].

## 5. Conclusion and future directions

PADI6 is an abundant and vital protein during early embryonic development. Despite this, the molecular mechanisms underlying its function are not clear. Several outstanding questions must be answered to understand its fundamental role in development. Most importantly, is PADI6 catalytically active? If so, what is its substrate profile and what are the activating and regulatory factors? PADI2 has recently been proposed to have possible non-catalytic functions through PPIs and thus dissecting a possible catalytic function from other non-catalytic regulatory or scaffolding functions could be necessary [89]. In line with this, does PADI6 interact with the SCMC and what is the function of this interaction? What other proteins does PADI6 interact with in the early embryo? Regarding the phenotypes observed in women, how is PADI6 involved in maternal imprinting and DNA methylation? Elucidating the structural and functional significance of the lack of conservation in PADI6 will provide valuable insight into its function in early embryo development. Towards this, a high-resolution crystal structure of PADI6 will be invaluable for deciphering its functional differences from the other PADIs. More general questions that must be answered and to which an understanding of PADI6 biology might contribute include further elucidating the molecular mechanisms of EGA and the function of the CPLs.

The restriction of PADI6 to the early embryo and the lethality of its absence make *in vivo* analysis of its function in early embryonic development difficult. Low input sequencing techniques such as single-cell RNA-sequencing have been applied previously to studying protein function in the early embryo and could be an option for interrogating the function of PADI6 throughout development [90]. Moving beyond RNA-sequencing though, in the past it has often been unfeasible to gather enough material to produce meaningful datasets from less sensitive techniques such as proteomics and bisulfite sequencing. Recently, single-cell bisulfite and proteomic strategies have been reported and have potential to be highly valuable for the study of early embryonic development in the future [91–95]. The restriction to and abundance of PADI6 in the oocyte and early embryo do hold some clinical advantages; for example, recent studies have highlighted the potential to use PADI6 as a novel marker for oocyte and embryo quality as well as ovarian reserve in women receiving assisted reproductive technologies [96,97].

The difficulties in studying PADI6 are further compounded by challenges relating to studying citrullination itself. The similarity between citrulline and its precursor arginine can make its interrogation by common techniques such as mass spectrometry or immunochemistry problematic. In the absence of good pan-citrulline antibodies, an alternative strategy has been either to raise pan-antibodies against chemically modified citrulline or antibodies against specific citrullinated sequences [44,56,98,99]. While powerful approaches, in the context of PADI6, the lack of known substrates prevents the rational raising of sequence-specific antibodies and the sensitivity of the antibodies to chemically modified citrulline is likely insufficient in the embryo context. Furthermore, the difficulty in producing inhibitors that are selective for individual PADI family members makes selective probing of PADI function even more challenging. It is therefore of the utmost importance to develop novel tools and strategies for probing citrulline and PADI function in a proteome-wide cellular context. Towards this, Thompson and co-workers have developed a phenyl–glyoxal-based chemical probe that can be used for proteome-wide labelling and identification of citrullinated proteins [100,101].

The processes governing early embryo development and the activation of the embryo's genome are not fully understood. With 12–15% of reproductive-aged couples (over 48 million couples) suffering from infertility worldwide, for up to 15% of whom the causative factor remains unknown [102], a better understanding of this process is critical. To achieve this, an understanding of the function of key proteins in early development will be essential [103]. Given the abundance and clear importance of PADI6 during these early stages, elucidating its molecular mechanisms will not only reveal the full function of PADI6, but it should also provide valuable insight into early embryo development as a whole.

## Data Availability

This article has no additional data.
